# F79. ATTRIBUTION OF INTENTIONS IN PATIENTS WITH SCHIZOPHRENIA SPECTRUM DISORDERS WITH PERSECUTORY DELUSIONS

**DOI:** 10.1093/schbul/sby017.610

**Published:** 2018-04-01

**Authors:** Michal Hajdúk, Lucia Pavelková, Peter Ohrablo, Veronika Petrušová, Anton Heretik, Ľubica Forgáčová

**Affiliations:** 1 Comenius University; 2 Slovak Medical University UHB

## Abstract

**Background:**

Social cognitive deficits are considered hallmark features of schizophrenia spectrum disorders. Consistent patterns of relationships have been established between theory of mind impairment and severity of negative symptoms. Some studies have suggested that patients, specifically those with persecutory delusion, can over attribute intentions. Difficulties in theory of mind in patients with schizophrenia can vary between hypo and hyper – mentalization depending on the level of symptoms. The aim of the study was to test model which proposed hypo -mentalization vs. hyper - mentalization deficit in patients with schizophrenia spectrum disorders with persecutory delusions.

**Methods:**

40 patients diagnosed with schizophrenia spectrum disorder, 19 patients with anxiety, affective and personality disorders without persecutory delusions, and 28 healthy controls were enrolled in the study. Diagnoses were established according to ICD-10 criteria. Animation Task was used for theory of mind assessment. Task consists of 12 videos (moving triangles) with three types of stimuli (random, goal-directed and theory of mind – condition). Stimuli were presented in fixed, random order before symptom assessment. Participants were asked to describe content of videos, and the degree of intentionality and appropriateness was evaluated by two raters according to task`s manual. Mutual rating of raters was used in the present analysis. Brief Psychiatric Rating Scale was used for assessment of symptoms severity.

**Results:**

A repeated measures ANOVA with stimuli type as within-factor and group as between-factor revealed significant effect of Stimuli type (F= 171.585, p < .001), and interaction of factors (F = 5.401, p = .001) on rating of intentionality. Group effect was not significant (F= .836, p = .437). Patients with schizophrenia had significantly lower ratings of intentionality in theory of mind condition, specifically. A second repeated measures ANOVA analyzed differences in levels of appropriateness. Results revealed significant effect of stimuli type (F= 12.698, p < .001), group (F= 6.966, p = .002) and interaction of factors (F = 3.211, p = .020). Responses of patients with schizophrenia were less appropriate than controls in goal-directed and theory of mind condition compared to the random condition. Severity of negative symptoms was associate with lower level of intentionality in random condition. Hostility and suspiciousness were associated with higher level of intentionality in goal directed (rs=.330, p=.037) and theory of mind conditions (rs=.348, p=.028). Severity of suspiciousness was moderately to strongly associated with appropriateness of descriptions in all conditions (rs from -.423 to -.517).

**Discussion:**

Results of study highlighted importance of distinguishing between hyper- and hypo-mentalization in patients with schizophrenia as specific impairments were associated with positive and negative symptoms, respectively. Over attribution of intentions to random movement was moderately associated with paranoid symptoms. Patients provided less appropriate descriptions which was associated with higher level of suspiciousness. Implications for development, maintenance treatment of persecutory delusions will be discussed.

Research was supported by Psychiatric Society of Slovak Medical Agency – grant no: 02/2015

